# Regulatory role of heme oxygenase-1 in silica-induced lung injury

**DOI:** 10.1186/s12931-018-0852-6

**Published:** 2018-08-01

**Authors:** Kentaro Nakashima, Takashi Sato, Suguru Shigemori, Takeshi Shimosato, Masaharu Shinkai, Takeshi Kaneko

**Affiliations:** 1 Department of Pulmonology, Yokohama City University Graduate School of Medicine, 3-9 Fukuura, Kanazawa-ku, Yokohama, Kanagawa 2360004 Japan; 2 Matebologenomics Core, Transborder Medical Research Center, University of Tsukuba, Ibaraki, Japan; 3 Department of Interdisciplinary Genome Sciences and Cell Metabolism, Institute for Biomedical Sciences, Shinshu University, Nagano, Japan

**Keywords:** Silicosis, Heme oxygenase-1, Antioxidant, Extracellular signal-regulated kinase, Reactive oxygen species

## Abstract

**Background:**

Silicosis, a progressive inflammatory lung disease attributed mainly to occupational exposure to silica dust, shows loss of lung function even after cessation of exposure. In addition to conventional evaluation methods such as chest X-ray, computed tomography, and spirometry, we identified heme oxygenase (HO)-1, an inducible antioxidant, as a potential biomarker to identify at-risk patients. We found that HO-1 was critical in attenuating the disease progression of silicosis; however, the key signaling pathway has not yet been elucidated. Here, we report the critical pathway after silica exposure, focusing on the role of silica-derived reactive oxygen species (ROS) signaling and its attenuation, which is mediated by HO-1 induction, in vivo and in vitro.

**Methods:**

Normal bronchial epithelial cells and a macrophage cell line, as well as a murine silicosis model generated by intratracheal administration of 2.5 mg of crystalline silica, were used in this study. The pathways activated in response to silica exposure, including the mitogen-activated protein kinase (MAPK) signaling pathway, were examined and compared with or without super-induction of HO-1.

**Results:**

The murine silicosis model was first assessed for the evaluation of activated pathways after silica exposure, focusing on ROS-MAPK activation. In the murine model, increased expression of HO-1 in the lungs was observed after silica-instillation. Moreover, silica-medicated activation of extracellular signal-regulated kinase (ERK) in the lungs was attenuated in response to silica-induced HO-1 upregulation. Activation of other MAPKs, such as p38 and c-Jun N-terminal kinase pathways, after silica exposure was not significantly different irrespective of HO-1 induction. Further in vitro studies showed that 1) silica-induced HO-1 was significantly attenuated by inhibiting ERK activation, and 2) carbon monoxide and bilirubin as final byproducts of HO-1 could inhibit ERK activation. Taken together, silica-induced HO-1 upregulation was mediated by ERK activation, and HO-1 further regulates ERK activation via its final byproducts, carbon monoxide and bilirubin.

**Conclusions:**

This is the first study to demonstrate the regulatory role of HO-1 in silicosis. This finding could contribute to the development of a treatment strategy of monitoring HO-1 levels as a marker of therapeutic intervention.

**Electronic supplementary material:**

The online version of this article (10.1186/s12931-018-0852-6) contains supplementary material, which is available to authorized users.

## Background

Silicosis is an irreversible and incurable lung disease caused by the inhalation of crystalline silica particle-containing dust, and is one of the most important occupational diseases in the world [1]. Silicosis typically occurs in workers exposed to abrasive blasting with sand, jackhammering, silica milling, rock drilling, and tunneling, and is thus especially common in industrializing countries [2]. Despite vigorous efforts to prevent exposure by dust control measures, silicosis continues to be a global problem [3]. In China, 6000 new cases of silicosis are reported annually and 24,000 silicosis patients die per year due to disease progression [1]. The most clinically important health impact from silicosis is lung function impairment due to massive inflammation, especially in acute silicosis, followed by destruction of the lung tissue, which is characterized by granulomatous and fibrotic lesions, in chronic silicosis [4, 5]. Regarding the mechanism of silicosis development and progression, various hypotheses such as cytotoxicity, oxidative stress, stimulation of inflammatory responses and induction of fibrosis have been proposed [6]. Elucidation of the molecular mechanisms triggered by exposure to silica may contribute to establish useful clinical markers and develop novel therapeutic strategies.

Crystalline silica produces reactive oxygen species (ROS), which play a major role in the development and progression of silicosis via the mitogen-activated protein kinase (MAPK) pathway [7–10]. ROS generated by silica exposure triggers phosphorylation of MAPK including extracellular signal-regulated kinases (ERK), c-Jun N-terminal kinases (JNK) or stress-activated protein kinases, and p38 kinases in vitro, leading to the activation of a number of transcriptional factors for genes involved in cellular proliferation, apoptosis, and inflammatory responses [11]. Therefore, targeting ROS with antioxidants is thought to be beneficial for silicosis treatment.

Heme oxygenase (HO)-1 is a rate-limiting enzyme that degrades heme into bilirubin, free iron, and carbon monoxide (CO) [12], and this HO-1 system including bilirubin and CO as byproducts represents a powerful cytoprotective antioxidant system [13, 14]. HO-1 is one of the proteins regulated by the MAPK signaling systems [15–18]. Conversely, Ryter et al. suggested that activated-MAPK signaling pathways, especially p38 MAPK, is modulated by CO, resulting in anti-inflammatory tissue protection [19]. Thus, the evidence suggests that there is cross-talk between the HO-1 metabolic system and the MAPK signaling systems.

Previously, we reported the association between HO-1 and silicosis in both murine model and human silicosis. Briefly, our findings are summarized as follows: 1) HO-1 was present in silicotic nodules in both murine and human lung samples, 2) HO-1 acts in a protective role by attenuating lung inflammation, 3) induced HO-1 in the lungs of silicosis subjects could be detected in serum and thus be monitored, and 4) low serum HO-1 could predict accelerated lung function decline in chronic silicosis [20, 21]. However, a key signaling pathway involved in the HO-1-mediated response to silica exposure has not yet been elucidated. Thus, the present study aimed to evaluate the critical pathway after silica exposure, focusing particularly on the role of silica-derived ROS signaling and its attenuation mediated by HO-1 induction.

## Methods

### Mice

Male BALB/c mice (6 weeks of age) were purchased from Japan SLC (Shizuoka, Japan), housed in light- and temperature-controlled rooms, and given free access to tap water and commercial laboratory chow. The mice were used for experiments following a one-week acclimation period.

### Murine silicosis model

The mice were anesthetized with ketamine (80 mg/kg; Sigma-Aldrich, St. Louis, MO, USA) or sodium thiopental (50 mg/kg Nembutal; Dainippon-Sumitomo Seiyaku. Co., Ltd., Osaka, Japan) and xylazine (10 mg/kg; Sigma). A 22-gauge cannula (Terumo, Tokyo, Japan) was inserted through the orotracheal route. Sterilized crystalline silica (Min-U-Sil-5, 100 mg/kg; US Silica, Berkeley Springs, WV, USA) in 100 μl of sterile saline was instilled into the trachea [21]. To induce HO-1 gene expression, 100 μmol/kg hemin (Sigma) was administered intraperitoneally 48, 24, and 0.5 h before silica administration. To inhibit ERK or HO-1 enzyme activity, U0126 (30 mg/kg; Merck, Darmstadt, Germany) or HO-1 inhibitors of either zinc protoporphyrin (ZnPP, 100 μmol/kg; Porphyrin Products, Logan, UT, USA) as a competitive HO-1 inhibitor or ketoconazole (KTZ, 100 μmol/kg; Sigma) as a selective HO-1 inhibitor was administered intraperitoneally in the same manner, respectively [22–25]. The lungs were removed 1, 2, 3, 7, and 14 days after silica instillation.

### Cell culture

The mouse macrophage-like cell line RAW264.7 (derived from BALB/c mice) from the American Type Culture Collection (ATCC; Rockville, MD, USA), and bronchial epithelial cells, transformed human bronchial epithelial cells 16HBE, kindly provided by D.C. Gruenert (Gene Therapy Center, University of California, CA, USA) were used [26]. The cells were cultured in DMEM (Sigma) supplemented with 10% FCS (Equitech-Bio, Kerrville, TX, USA), 1% penicillin, and 1% streptomycin (Sigma) at 37 °C in 5% CO_2_. The cells were cultured to subconfluence in 6-well plates (Sumitomo, Osaka, Japan), then rendered quiescent in medium containing 0.5% FCS for another 1 day, followed by exposure of 0.1 or 0.5 mg/ml of sterilized silica for 24 h. In some experiments, the cells were pretreated with one of the following agents before silica treatment: 1 h with 50 or 100 μM of the ERK inhibitor, U0126 (Cell Signaling Technology, Danvers, MA, USA), 1 h with 25 mM or 50 mM tetramethylthiourea (TMTU; MP Biomedicals, Santa Ana, CA, USA), 16 h with 5 or 10 μM bilirubin (Sigma), 1 h with 20 or 50 μM RuCO (CO-releasing molecule; Sigma), 1 h with 100 or 200 μM hemin, or 1 h with 100 or 200 μM ZnPP. The cells were collected either 2–6 or 8–24 h after silica exposure and evaluated for ERK activation or HO-1 induction, respectively, in RAW264.7 or 16HBE cells.

### Immunoblotting analysis

The lung was homogenized in lysis buffer containing 0.25 M sucrose, 20 mM Tris-HCl (pH 7.4), and protease inhibitor (Sigma). After centrifugation (15,000×g for 10 min), the supernatants were collected as the cytoplasm fraction. The pellets were sonicated and further separated into the nuclear, mitochondrial, and microsomal fractions by multi-step centrifugations as previously described [27]. The nuclear fractions were resolved with nuclear extraction buffer containing 20 mM HEPES (pH 7.6), 20% glycerol, 500 mM NaCl, 1.5 mM MgCl_2_, 0.2 mM EDTA (pH 8.0), 1 mM DTT, 0.1% NP-40, and protease inhibitor. The protein concentration in each sample was determined by the Bradford method using the Bio-Rad Protein assay kit (Bio-Rad, Hercules, CA, USA). After 5 min boiling of 10 μg microsomal extraction or 20 μg nuclear extraction with an equal volume of sample buffer, the samples were run on 4–20% gradient polyacrylamide gels (Daiichi Kagaku, Tokyo, Japan) for electrophoresis. Then, the proteins in the membranes were transferred onto Immobilon polyvinylidene difluoride membranes (Millipore, St. Louis, MO, USA). The membranes were blocked in blocking buffer (5% nonfat dry milk in Tris-buffered saline, pH 7.5, 0.05% (*v*/v) Tween20 (TBST)) for 1 h at room temperature, and then incubated with the relevant antibodies for 1 h at room temperature. The following rabbit polyclonal antibodies were used: phospho-ERK1/2 (1:1000 dilution), phospho-JNK (1:1000 dilution), phospho-p38 (1:1000 dilution), ERK1/2 (1:1000 dilution) from Cell Signaling Technology, and HO-1 (OSA-111; ENZO Life Sciences, Farmingdale, NY, USA; 1:1000 dilution). After washing with TBST, the blots were incubated with the appropriate peroxidase-conjugated secondary antibody (1:5000 dilution) for 1 h at room temperature and developed using an enhanced chemiluminescence system (ECL; Amersham, Buck, UK) and Hyperfilm (Amersham). The blotted protein was quantified densitometrically with ImageJ image analysis and image processing software (Version 1.51; National Institutes of Health Image Engineering, Bethesda, MD, USA). β-Actin (A1978; Sigma; 1:10000 dilution) was used as a positive control.

### Statistical analysis

Data were analyzed using a statistical software package (ystat2004.xls; Igaku Tosho Shuppan, Tokyo, Japan) or MedCalc version 18 software (Mariakerke, Belgium). Densitometric data were analyzed using either a Student’s t-test or one-way analysis of variance with the post hoc Student-Newman-Keuls test. Values are expressed as the mean ± SD. Differences with a *P* value of < 0.05 were considered significant.

## Results

### HO-1 expression in murine silicosis

**Fig. 1:**
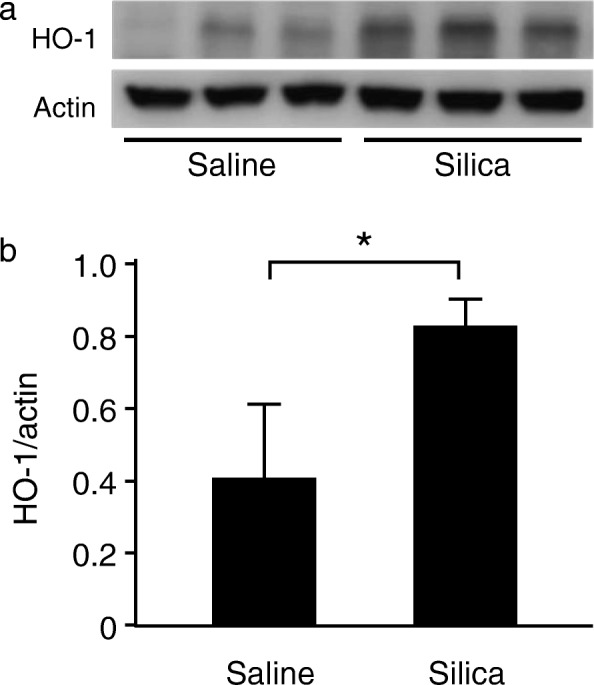
HO-1 expression in the lungs in murine silicosis. Mice were instilled with 2.5 mg of silica particles. **a** The lung samples collected 2 days after silica instillation were subjected to SDS-PAGE (10%; 15 μg of total protein/lane) and analyzed by Western blotting using anti-HO-1 and anti-actin antibodies. **b** Densitometric analysis of band intensity representing the mean ± SD level of HO-1 protein relative to actin (*n* = 3/group). The ratio of HO-1/actin was significantly increased in the lungs in murine silicosis compared to the saline control (Student’s t-test). * *P* < 0.05

To confirm the effect of silica on HO-1 activation, we studied a murine model of silicosis. After acclimation, crystalline silica was administered intratracheally to BALB/c mice under anesthesia. Immunoblotting analysis was performed on lung homogenates of silicosis mice. The expression levels of HO-1 protein in the lungs were significantly increased 2 days after silica instillation compared with those from control mice (Fig. 1a/b). These observations are consistent with the previous findings [21].

### HO-1 induction suppresses ERK1/2 activation in murine silicosis

**Fig. 2:**
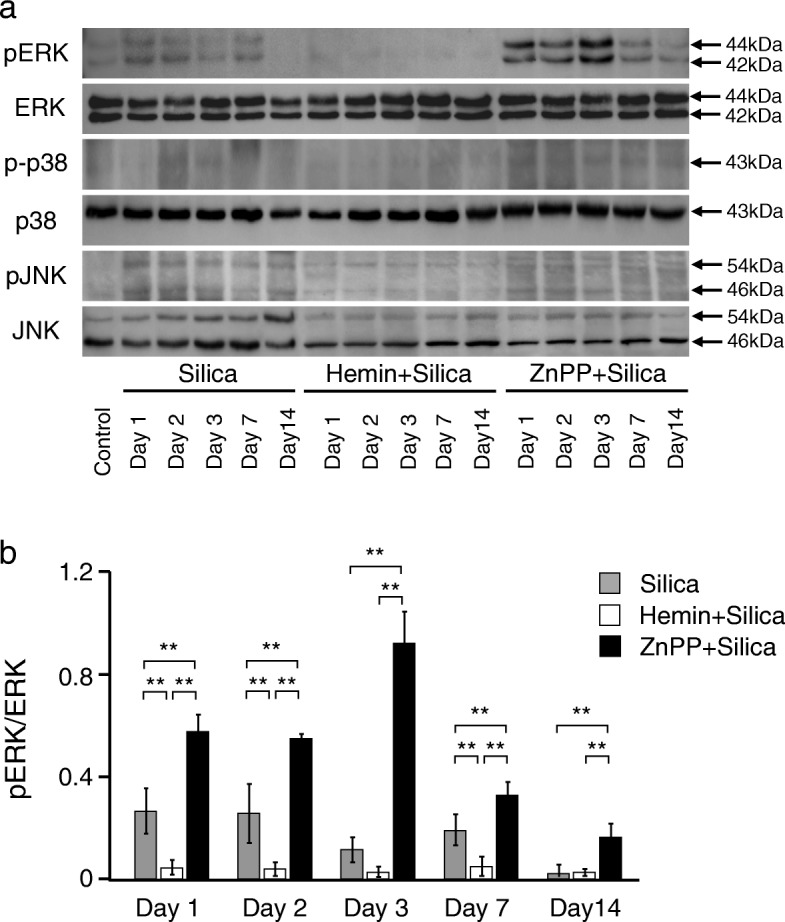
Effects of HO-1 inducer/inhibitor on MAPK in the lungs in murine silicosis. **a** Expression levels of phospho-ERK (pERK), phospho-p38 (p-p38), and phospho-JNK (pJNK) were determined in 20 μg of lung nuclear extractions by using relevant antibodies. pERK was upregulated 1 day after silica instillation without pretreatment. pERK was hardly detected following pretreatment with hemin, whereas the expression was enhanced by ZnPP. There were no differences in expression levels of p-p38 and pJNK with or without pretreatment with either inducer or inhibitor of HO-1. **b** Densitometric analysis of band intensity representing the mean ± SD level of pERK to ERK (*n* = 4/group). The ratio of pERK/ERK was significantly decreased at days 1, 2, and 7 in the lungs in murine silicosis pretreated with HO-1 inducer compared to no pretreatment group (silica alone), whereas those pretreated with HO-1 inhibitor showed a sustained increase in the pERK/ERK ratio over time. Densitometric data were analyzed by one-way analysis of variance with the post hoc Student-Newman-Keuls test. ** *P* < 0.01

**Fig. 3:**
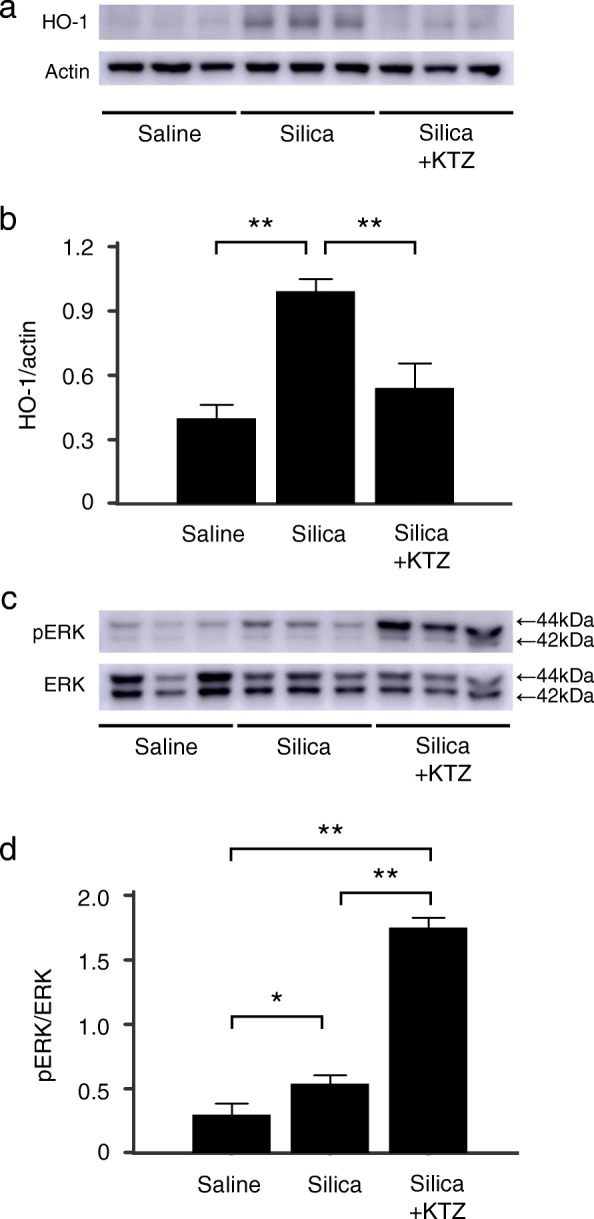
Effect of ketoconazole on HO-1 induction and ERK activation in the lungs in murine silicosis. Mice were administered intraperitoneally with the HO-1 selective inhibitor ketoconazole (KTZ) at 48, 24, and 0.5 h before 2.5 mg of silica particles instillation. **a** Lung samples collected 2 days after silica instillation were analyzed as described in Fig. 1. **b** Densitometric analysis of band intensity representing the mean ± SD level of HO-1 protein relative to actin (n = 3/group). The ratio of HO-1/actin was significantly decreased in the lungs in murine silicosis pretreated with KTZ compared to the no pretreatment group (silica alone). **c** Lung samples collected 2 days after silica instillation were analyzed as described in Fig. 2. **d** Densitometric analysis of band intensity representing the mean ± SD level of pERK to ERK (n = 3/group). The ratio of pERK/ERK was significantly increased in the lungs in murine silicosis pretreated with KTZ compared to the no pretreatment group (silica alone). *, *P* < 0.05; **, *P* < 0.01

MAPK systems are known to be major factors affecting the disease progression of silicosis [7, 28], with HO-1 as a newly recognized factor [20, 21]. However, the relationship between MAPK systems and HO-1 after silica exposure has not yet been elucidated. To investigate the key signaling pathway involved in the HO-1-mediated response to silica exposure, we first examined phosphorylated MAPK proteins of ERK, p38, and JNK in lungs from murine silicosis. Mice were divided into four groups: 1) pretreated with hemin, an inducer of HO-1, then treated with silica, 2) pretreated with ZnPP, a competitive inhibitor of HO-1, then treated with silica, 3) treated with silica alone, and 4) treated with saline alone. Silica-induced MAPK activation was examined and compared with or without pretreatment with the HO-1 inducer and inhibitor. As shown in Fig. 2a, expression levels of phosphorylated ERK in the lungs were upregulated 1 day after silica exposure, and then gradually decreased. In contrast, expression levels of phosphorylated p38 and JNK were continually increased after silica exposure and were not altered with or without pretreatments of either hemin or ZnPP (Fig. 2a). Most importantly, the expression level of phosphorylated ERK was significantly decreased by pretreatment with hemin, but was significantly increased by pretreatment with ZnPP (Fig. 2a/b). These results suggest that the beneficial effects of HO-1 induction in murine silicosis are associated with the ERK signaling pathway. Consistent with this, mice subjected to intraperitoneal administration of the ERK inhibitor, U0126, 2 h before and 6 h after silica administration showed attenuated HO-1 induction in the lungs (Additional file 1). These results indicated the feedback system between HO-1 and ERK activation. To determine the specific involvement of HO-1, further experiments using KTZ as a selective inhibitor of HO-1 were performed. Mice were administered KTZ intraperitoneally 48, 24, and 0.5 h before silica administration. As shown in Fig. 3a/b, pretreatment with KTZ significantly inhibited HO-1 induction in the lungs 2 days after silica instillation, and the level of activated ERK was significantly higher in silicosis mice pretreated with KTZ compared to those without (Fig. 3c/d). These results are comparable to those using the competitive HO-1 inhibitor ZnPP (Fig. 2). Taken together, these data suggested that HO-1, rather than another heme oxygenase, negatively regulates phosphorylation of ERK.

### Silica-mediated ERK activation and HO-1 induction in vitro

**Fig. 4:**
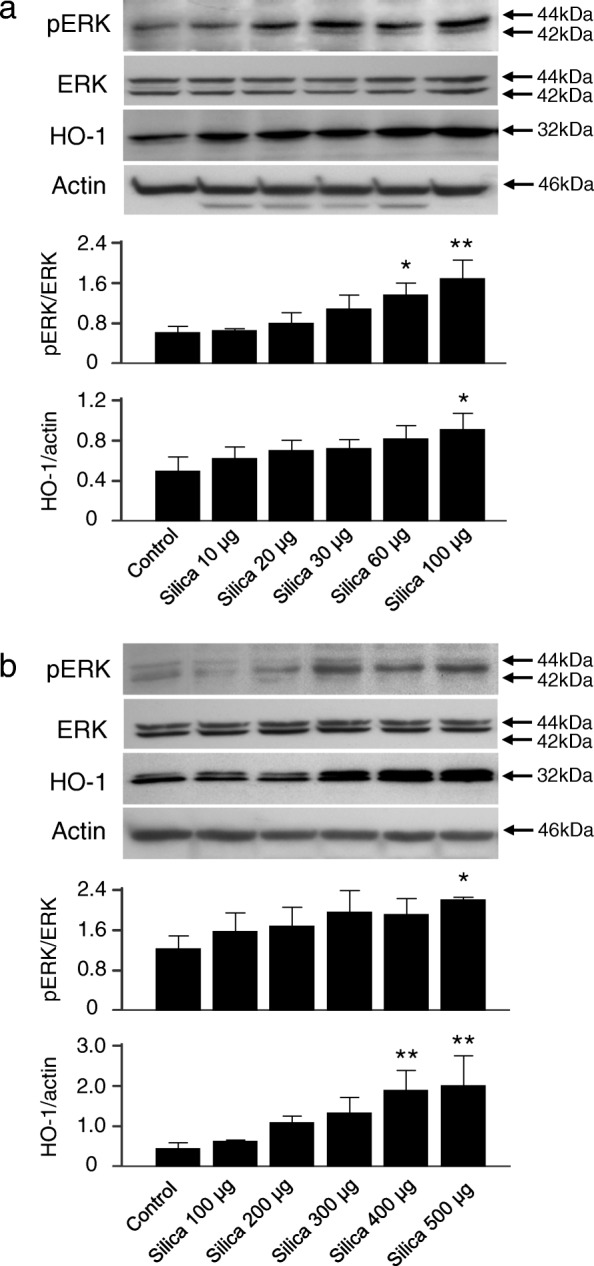
Silica induced pERK and HO-1 in RAW264.7 and 16HBE cells. Immunoblotting analysis revealed that silica induced pERK upregulation and HO-1 expression in a dose-dependent manner. Cells were exposed to vehicle (Control) or silica at the indicated concentrations for 2 h (pERK) or 8 h (HO-1) in (**a**) RAW264.7 cells or for 6 h (pERK) or 24 h (HO-1) in (**b**) 16HBE cells, respectively. Representative immunoblot images from three independent experiments are shown. Densitometric analysis of band intensity representing the mean ± SD level of pERK to ERK or HO-1 to actin from three independent experiments * *P* < 0.05; ** *P* < 0.01 vs control

As shown in our previous report, silica-induced HO-1 upregulation was evident in macrophages and bronchial epithelial cells in silicotic lungs from both mice and humans [21]. Thus, to further investigate the relationship between HO-1 and its products with the ERK pathway, the protein expression of phosphorylated ERK and HO-1 in silica-stimulated RAW264.7, a macrophage cell line (Fig. 4a), and 16HBE, a bronchial epithelial cell line (Fig. 4b), was analyzed by immunoblotting. As shown in Fig. 4a/b, phosphorylated ERK expression, indicating ERK activation, was obvious when more than 30 μg or 300 μg of silica was administered in RAW264.7 or 16HBE cells, respectively. HO-1 expressions were increased in a dose-dependent manner in both cell lines exposed to silica (Fig. 4a/b). For subsequent studies, the dose of silica was set at 100 or 500 μg for either RAW264.7 or 16HBE cells, concentrations which produced significant ERK activation and HO-1 induction, respectively (Fig. 4a/b lower panel).

### Silica-derived ROS induces HO-1 via ERK1/2 activation in vitro

**Fig. 5:**
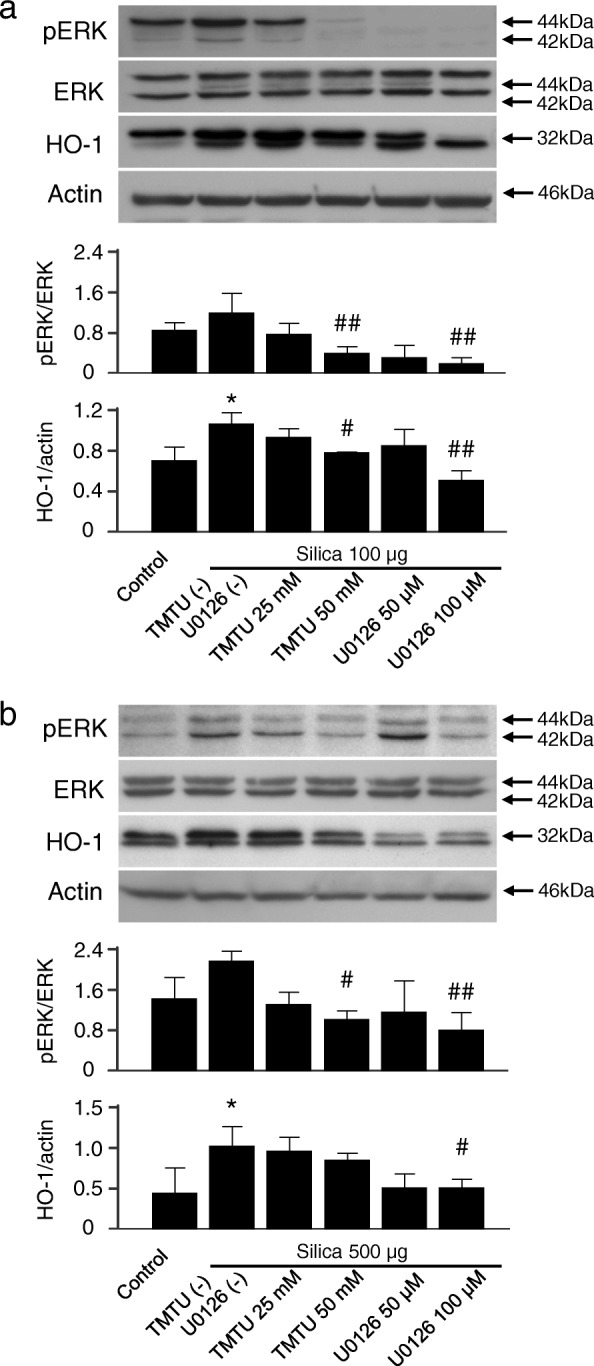
Effect of ERK inhibition on silica-induced HO-1 upregulation in RAW264.7 and 16HBE cells. Immunoblotting analysis showed that ERK inactivation by radical scavenger or ERK inhibition by MEK/ERK inhibitor suppressed silica-induced HO-1 upregulation in (**a**) RAW264.7 cells and (**b**) 16HBE cells. Cells were pretreated with TMTU as a hydroxyl radical scavenger or U0126 as a MEK1/2 inhibitor 1 h before silica exposure at the indicated concentrations and incubated as described in Fig. 4. Representative immunoblot images from three independent experiments are shown. Densitometric analysis of band intensity representing the mean ± SD level of pERK to ERK or HO-1 to actin from three independent experiments. * *P* < 0.05 vs control. ^#^ *P* < 0.05; ^##^ *P* < 0.01 vs silica only

Since ROS are thought to play a major role in the pathogenicity of crystalline silica [8], we next evaluated the relationship between ROS and HO-1. Cells were stimulated with silica in the presence of a hydroxyl radical scavenger, TMTU, and ERK inhibitor, U0126. As shown in Fig. 5, either TMTU or U0126 dose-dependently suppressed not only phosphorylation of ERK but also HO-1 induction in both RAW264.7 (Fig. 5a) and 16HBE (Fig. 5b) cells exposed to silica. These data suggest that ROS blocking by the radical scavenger led to the suppression of ERK activation, resulting in decreased HO-1 expression.

### HO-1 expression levels regulate silica-induced ERK1/2 activation in vitro

**Fig. 6:**
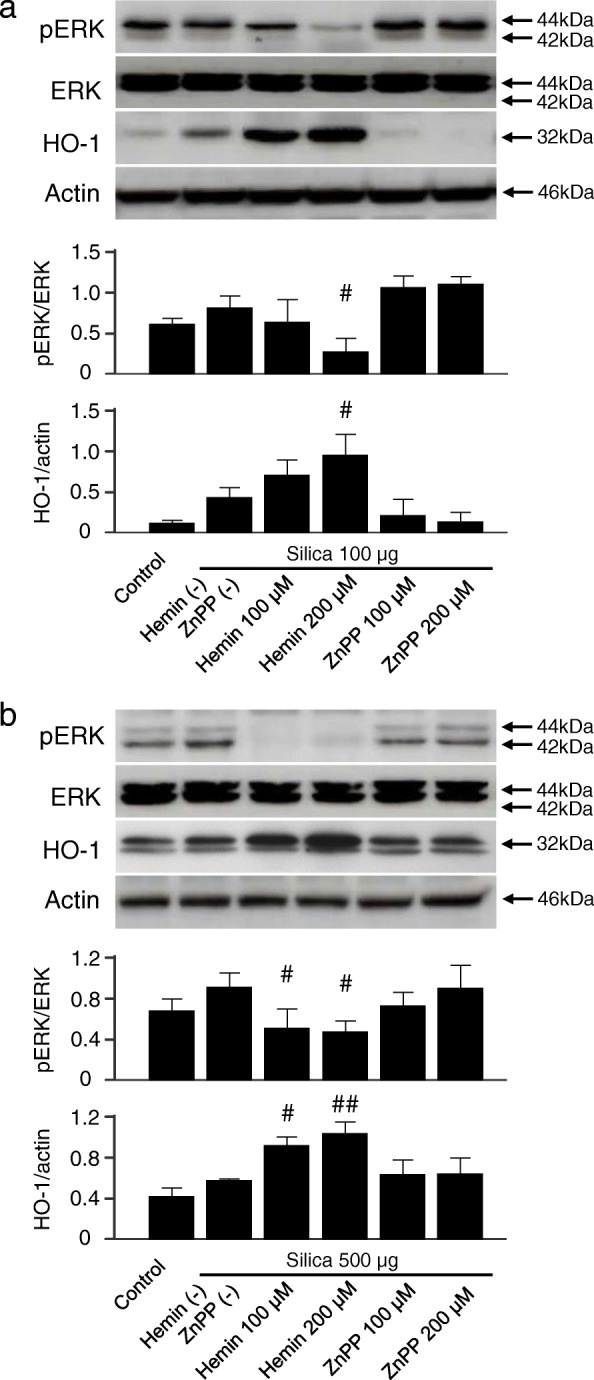
Effect of HO-1 inducer/inhibitor on silica-induced ERK activation in RAW264.7 and 16HBE cells. Immunoblotting analysis showed that HO-1 inducer/inhibitor modulated silica-induced ERK activation in (**a**) RAW264.7 cells and (**b**) 16HBE cells. Cells were pretreated with hemin (HO-1 inducer) or ZnPP (HO-1 inhibitor) 1 h before silica exposure at the indicated concentrations and incubated as described in Fig. 4. Representative immunoblot images from three independent experiments are shown. Densitometric analysis of band intensity representing the mean ± SD level of pERK to ERK or HO-1 to actin from three independent experiments. ^#^ *P* < 0.05; ^##^ *P* < 0.01 vs silica only

As shown in the result from the silicosis model (Fig. 2), pretreatment with hemin could effectively pre-induce HO-1 in the lungs, whereas pretreatment with ZnPP suppressed HO-1 induction after silica administration. Similarly, Fig. 6a/b shows expression levels of phosphorylated ERK were suppressed by pretreatment with hemin, but not with ZnPP in both RAW264.7 and 16HBE cells. These results indicated that HO-1 negatively regulates phosphorylation of ERK, which is in agreement with that observed in murine silicosis (Fig. 2).

### HO-1-derived metabolites suppress ERK1/2 activation in vitro

**Fig. 7:**
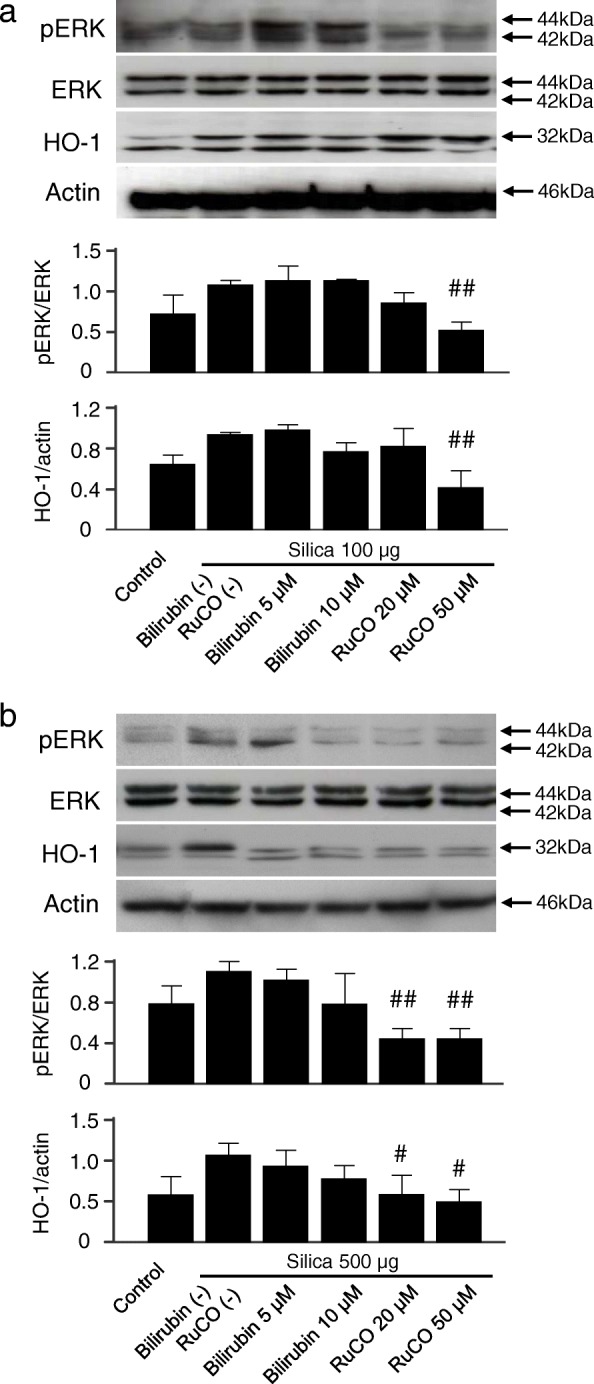
Effect of HO-1 byproducts on silica-induced pERK and HO-1 upregulation in RAW264.7 and 16HBE cells. Immunoblotting analysis showed that HO-1 byproducts, bilirubin and CO, suppress silica-induced ERK activation and subsequent HO-1 upregulation in (**a**) RAW264.7 cells and (**b**) 16HBE cells. Cells were pretreated with bilirubin or RuCO (CO releasing molecule) 1 h before silica exposure at the indicated concentrations and incubated as described in Fig. 4. Representative immunoblot images from three independent experiments are shown. Densitometric analysis of band intensity representing the mean ± SD level of pERK to ERK or HO-1 to actin from three independent experiments. ^#^ *P* < 0.05; ^##^ *P* < 0.01 vs silica only

Based on the results so far, the ROS-ERK pathway could be a key mediator of silica-induced HO-1 upregulation. Finally, we further investigated which of the heme degradation products (bilirubin or CO) was responsible for regulation of the ERK pathway, because hemin-mediated pre-induction of HO-1 could suppress ERK activation (Figs. 2 and 6a/b). Cells were exposed to bilirubin, an antioxidant, or RuCO, a CO-releasing molecule, before silica treatment, and the results showed that CO especially suppressed phosphorylated ERK expression (Fig. 7a/b). These results are consistent with previous reports describing the effect of HO-1 (and its by-product) on modulating ERK [29–31]. Interestingly, HO-1 expression was associated dose-dependently with pretreatment of either bilirubin or RuCO (Fig. 7a/b). As the ERK pathway has been shown to be involved in the transcription of HO-1 [32], the present results indicate that the exposure to silica particles triggered hydroxyl radical generation and induced HO-1 through activation of the ERK pathway (Figs. 4a/b and 5a/b). Furthermore, high HO-1 expression could regulate silica-mediated ERK activation by heme degradation products, CO and bilirubin (Figs. 6a/b and 7a/b).

## Discussion

**Fig. 8:**
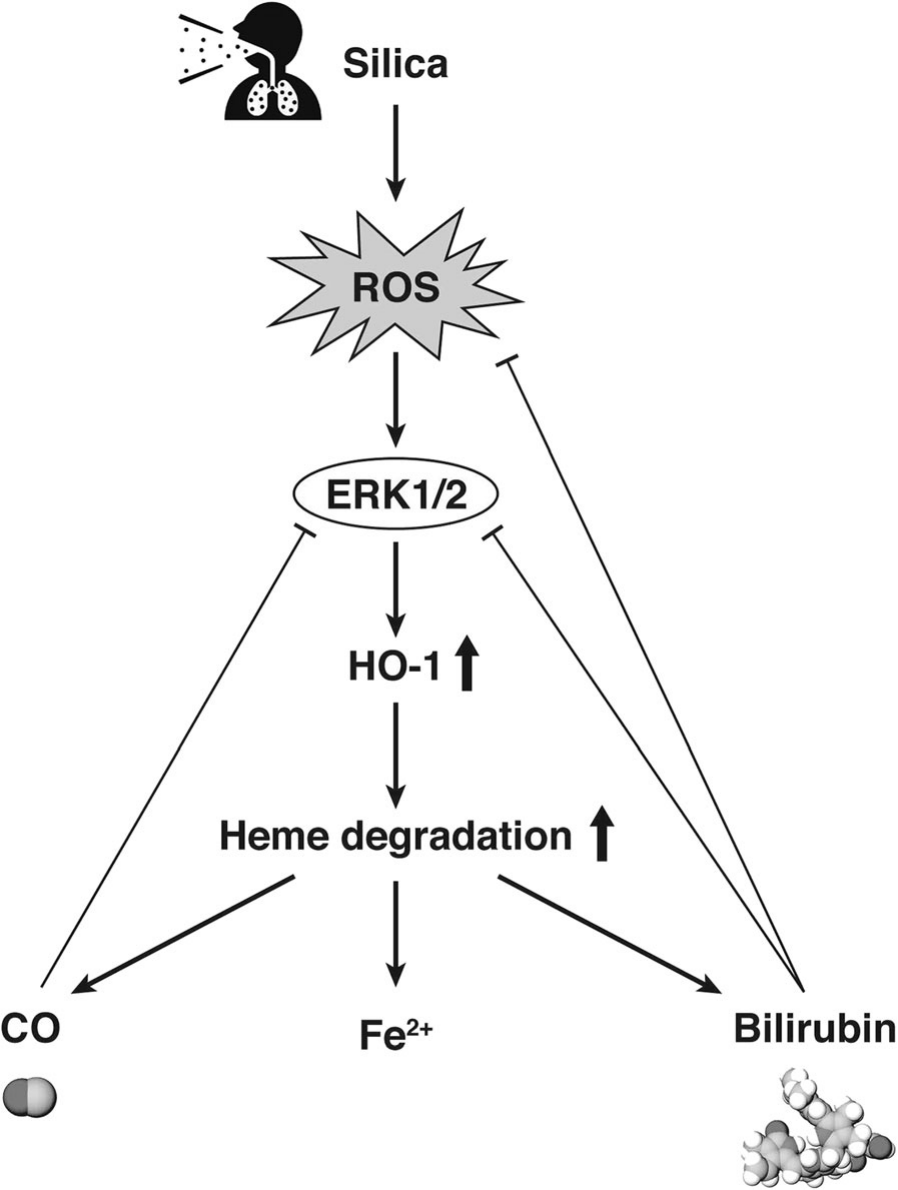
Schematic diagram of the regulatory pathway involved in silica-induced HO-1 expression. Exposure to silica particles produces ROS, which in turn initiates the activation of ERK and ultimately induces HO-1 expression. Silica-derived ROS are scavenged by the heme degradation product bilirubin. Silica-induced ERK activation is attenuated by the heme degradation products CO and bilirubin

We have reported that the anti-oxidant protein HO-1 could be a key monitoring parameter as well as a potential therapeutic for silicosis [20, 21]. The present study demonstrated for the first time that HO-1 could have a regulatory role in silica-mediated ERK activation both in vivo and in vitro. In the murine model, phosphorylated ERK expression in the lungs was elevated after exposure to crystalline silica, while ERK activation was attenuated in response to silica-induced HO-1 expression or pretreatment with HO-1 inducer (Figs. 1 and 2). Activation of other MAPKs, such as the p38 and JNK pathways, after silica exposure was not significantly different with or without HO-1 induction (Fig. 2). These results suggested that the silica-ERK-HO-1 pathway might be regulated via a negative feedback loop, as illustrated in the schematic diagram shown in Fig. 8. Thus, HO-1 induction might be a novel therapeutic strategy to control excess activation of ERK after silica exposure (Fig. 8). This strategy was further assessed in another experiment using either U0126 as a specific inhibitor of ERK1/2 or KTZ as a selective inhibitor of HO-1 to determine the association between ERK and HO-1 following silica exposure (Additional file 1 and Fig. 3). These results clearly indicated that silica-induced HO-1 was a key in controlling ERK activation, as evidenced by HO-1 competitive/selective inhibitors augmenting ERK activation after silica exposure (Figs. 2 and 3). Therefore, we attempted to elucidate the precise mechanism in vitro.

There is accumulating evidence that HO-1 plays a protective role in the progression of various disease [33]. Besides our previous silicosis studies, there have been many reports showing that HO-1 induction is effective for lipopolysaccharide-induced acute lung injury [23], influenza virus-induced lung injury [34], and *Pseudomonas aeruginosa*-induced lung inflammation in murine models [35].

MAPK has been shown to regulate a variety of cellular functions including cell growth, proliferation, differentiation, migration, and apoptosis [36]. MAPK activation has been demonstrated in mouse epithelial cells [7, 37, 38], macrophages [39], and fibroblasts [40] in response to crystalline silica. In the present study, we confirmed that silica induced the activation of MAPK pathways (ERK, JNK, and p38) in vivo. Of these, we clarified that ERK plays an important role in silica-induced HO-1 expression (Figs. 4 and 5). The results seem reasonable in light of a report that ERK is involved in translocation of the transcription factor NF-E2-related factor 2, which regulates induction of the HO-1 gene [32].

Production of ROS such as hydrogen peroxide following silica exposure is not only generated directly by the silica particles, but also by phagocytic cells attempting to digest silica particles, and ROS is one of the most important factors in the development of silicosis [8, 41]. For example, ROS-induced apoptosis of phagocytic macrophages releases large amounts of inflammatory mediators that induce tissue damage, which is associated with the fibrotic changes observed in silicosis [42, 43]. As shown in the present study, ROS also activates the MAPK/ERK signaling pathway (Fig. 5).

Accumulating evidence suggests that persistent activation of the ERK pathway is associated with pulmonary fibrosis and subsequent carcinogenesis in lung diseases [44, 45]. As aberrant activation of ERK leads to prolonged inflammation, dysregulation of proliferation and further malignancy development, the concept of negative feedback regulation of ERK or ERK inhibition seems more attractive for fibrosis prevention and cancer treatment [46, 47]. In this regard, the present study confirmed a negative feedback regulation of ERK activation by high induction of HO-1 and its metabolites (bilirubin and CO), which have powerful antioxidant properties derived from the silica-ERK-HO-1 axis (Figs. 6 and 7). This mechanism could exert prompt attenuation of the silica-induced lung inflammation and subsequent lung injury shown previously [21].

In the present study, it should be noted that 1) as ZnPP is a competitive but non-specific inhibitor of HO-1 [48], we performed the in vivo experiments using KTZ as a HO-1 selective inhibitor. However, since KTZ has been examined only in vivo, we plan to examine the in vitro effects of KTZ prior to future clinical use of HO-1 as a therapeutics [49], and 2) as HO-1 activity does not necessarily correlate with HO-1 mRNA or protein levels [50], we further plan to examine silica-induced HO-1 activity in macrophages and epithelial cells in the lung.

In human silicosis, persistent inflammation from the deposition of silica particles in the lungs could be modulated similarly by the negatively regulated mechanism of the silica-ERK-HO-1 axis (Fig. 8). Therefore, it should be proposed again that the monitoring of HO-1 as a diagnostic marker regulating silica-ERK signaling could be useful for slowing disease progression [20]. Recently, the HO-1 inducer hemin was examined in a phase IIB clinical trial of renal transplantation, and showed successful HO-1 upregulation [51]. Therefore, we would advocate the monitoring of HO-1 as a marker of therapeutic intervention. Thus, we have developed a tool for HO-1 supplementation by using genetically modified strains of lactic acid bacteria (LAB) that secrete bioactive HO-1 [52]. Further studies are planned to assess the effect of site, dose, and duration of HO-1-secreting LAB administration on the fibrosis and tumor development associated with silica exposure.

## Conclusions

In conclusion, our findings showed that the silica-mediated ROS-ERK signaling pathway leads to HO-1 induction, whose byproducts negatively regulate ROS-ERK signaling. Taken together with our earlier results, the strategy of HO-1 supplementation, which originates from monitoring serum HO-1 and ROS such as hydrogen peroxide in patients with silicosis, could offer a new treatment option by inhibiting the excess activation of ROS-ERK signaling.

## Additional file

Additional file 1: Effect of ERK inhibitor on HO-1 induction in the lungs in murine silicosis.Mice were administered intraperitoneally with the ERK inhibitor, U0126, 2 h before and 6 h after 2.5 mg of silica particles instillation. A) Lung samples collected 2 days after silica instillation were analyzed as described in Fig. 2. B) Densitometric analysis of band intensity representing the mean ± SD level of HO-1 protein relative to actin (*n* = 3/group). Although not significant, U0126 attenuated HO-1 induction after silica exposure. * *P* < 0.05; ** *P* < 0.01. (PDF 18 kb)

## Abbreviations

CO: Carbon monoxide
ERK: Extracellular signal-regulated kinases
HO-1: Heme oxygenase-1
JNK: c-Jun N-terminal kinases
KTZ: ketoconazole
MAPK: Mitogen-activated protein kinases
ROS: Reactive oxygen species.
TMTU: Tetramethylthiourea.
ZnPP: Zinc protoporphyrin.
