# Supplementary figures and images for: Regulatory role of heme oxygenase-1 in silica-induced lung injury
Source: Respir Res. 2018 Aug 1;19:144. doi: 10.1186/s12931-018-0852-6 (PMC6090697; doi:10.1186/s12931-018-0852-6)

Additional file 1

A

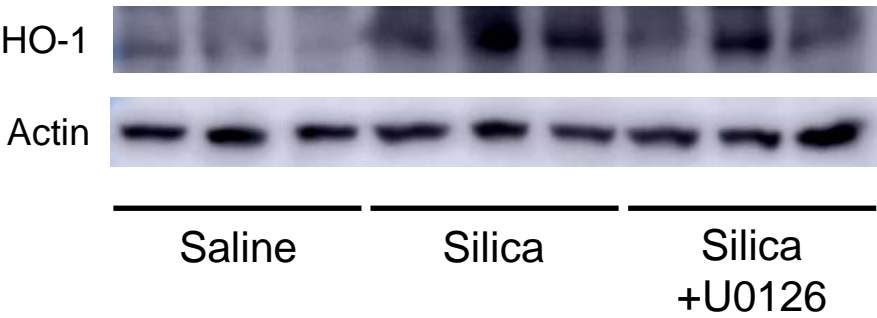

B

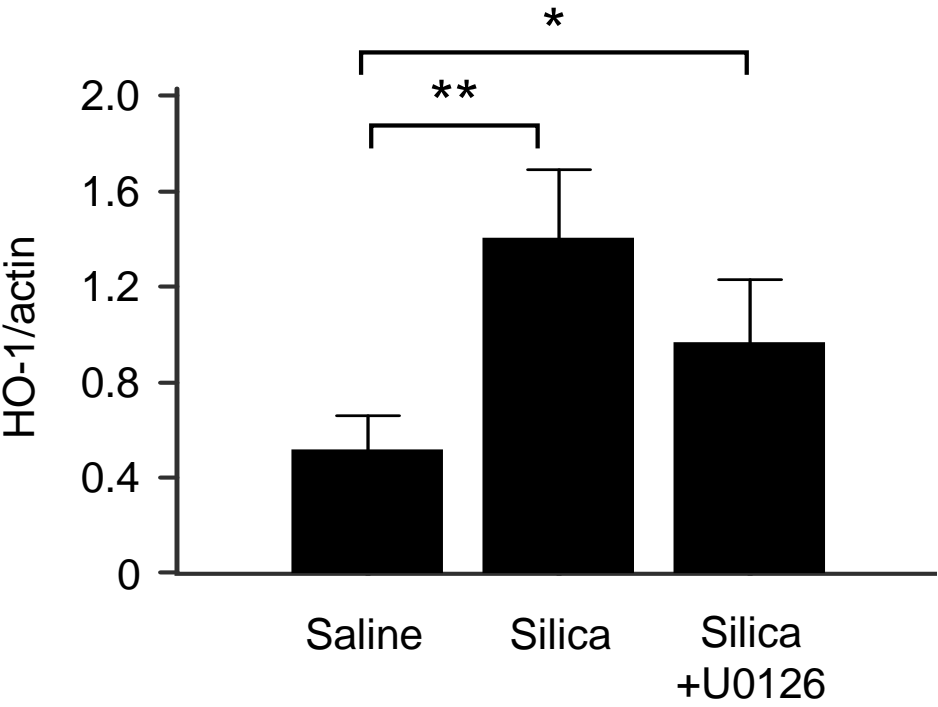

Supplement: Supplementary file 1 — Effect of ERK inhibitor on HO-1 induction in the lungs in murine silicosis.Mice were administered intraperitoneally with the ERK inhibitor, U0126, 2 h before and 6 h after 2.5 mg of silica particles instillation. A) Lung samples collected 2 days after silica instillation were analyzed as described in Fig. 2. B) Densitometric analysis of band intensity representing the mean ± SD level of HO-1 protein relative to actin (n = 3/group). Although not significant, U0126 attenuated HO-1 induction after silica exposure. * P < 0.05; ** P < 0.01. (PDF 18 kb) [file 12931_2018_852_MOESM1_ESM.pdf]
